# Frequency of empiric antibiotic de-escalation in an acute care hospital with an established Antimicrobial Stewardship Program

**DOI:** 10.1186/s12879-016-2080-3

**Published:** 2016-12-12

**Authors:** Peter Liu, Christopher Ohl, James Johnson, John Williamson, James Beardsley, Vera Luther

**Affiliations:** 1 Wake Forest School of Medicine, Medical Center Boulevard, Winston-Salem, NC 27157 USA; 2 Wake Forest School of Medicine, Section on Infectious Diseases, Medical Center Boulevard, Winston-Salem, NC 27157 USA; 3 Wake Forest Baptist Health, Medical Center Boulevard, Winston-Salem, NC 27157 USA

**Keywords:** Antibiotic de-escalation, Antibiotic stewardship, Antimicrobial stewardship program, Benchmark, Prospective audit and feedback

## Abstract

**Background:**

Expanding antimicrobial resistance patterns in the face of stagnant growth in novel antibiotic production underscores the importance of antibiotic stewardship in which de-escalation remains an integral component. We measured the frequency of antibiotic de-escalation in a tertiary care medical center with an established antimicrobial stewardship program to provide a plausible benchmark for de-escalation.

**Methods:**

A retrospective, observational study was performed by review of randomly selected electronic medical records of 240 patients who received simultaneous piperacillin/tazobactam and vancomycin from January to December 2011 at an 885-bed tertiary care medical center. Patient characteristics including antibiotic regimen, duration and indication, culture results, length of stay, and hospital mortality were evaluated. Antibiotic de-escalation was defined as the use of narrower spectrum antibiotics or the discontinuation of antibiotics after initiation of piperacillin/tazobactam and vancomycin therapy. Subjects dying within 72 h of antibiotic initiation were considered not de-escalated for subsequent analysis and were subtracted from the study population in determining a modified mortality rate.

**Results:**

The most commonly documented indications for piperacillin/tazobactam and vancomycin therapy were pneumonia and sepsis. Of the 240 patients studied, 151 (63%) had their antibiotic regimens de-escalated by 72 h. The proportion of patients de-escalated by 96 h with positive vs. negative cultures was similar, 71 and 72%, respectively. Median length of stay was 4 days shorter in de-escalated patients, and the difference in adjusted mortality was not significant (*p* = 0.82).

**Conclusions:**

The empiric antibiotic regimens of approximately two-thirds of patients were de-escalated by 72 h in an institution with a well-established antimicrobial stewardship program. While this study provides one plausible benchmark for antibiotic de-escalation, further studies, including evaluations of antibiotic appropriateness and patient outcomes, are needed to inform decisions on potential benchmarks for antibiotic de-escalation.

## Background

Antimicrobial resistance continues to increase at alarming rates. Moreover, the rate at which new, novel antimicrobial agents have been developed and approved for therapy of infections has considerably decreased. Consequently, efforts to promote judicious and appropriate use of antibiotics are of significant importance. Antibiotic de-escalation, in which broad, empiric antimicrobial therapy is either discontinued or replaced with a narrowed spectrum antibiotic, has been an area of increasing focus for decreasing or improving antibiotic use [1–4].

Studies have demonstrated that antibiotic de-escalation is safe and not associated with poorer outcomes [3–12]. Anticipated benefits of de-escalation include an improvement in antibiotic resistance profiles and a reduction of antibiotic-related adverse events. Furthermore, the current Infectious Diseases Society of America antimicrobial stewardship guidelines recommend the streamlining and de-escalation of empirical antimicrobial therapy based on culture results to more effectively target the causative pathogen, thereby resulting in decreased antimicrobial exposure and substantial cost savings [13].

The practice of antibiotic de-escalation is not standardized, and several barriers to antibiotic de-escalation have been reported. These barriers include complex sensitivity patterns of the offending organism, inconclusive microbiological data, colonization with multi-drug resistant pathogens, and receipt of previous antibiotic therapy [14]. Other studies have described factors related to providers’ clinical decision-making as key barriers to de-escalation. These factors include a lack of diagnostic facility, a lack of multidisciplinary collaboration, a lack of education among junior prescribers, and reluctance to de-escalate antibiotics in critically ill patients who are improving with broad spectrum therapy [15–17].

The frequency with which antibiotic regimens are de-escalated or should be de-escalated in the hospital setting is not well described. The few studies that are available suggest a variable frequency of antibiotic de-escalation which ranges from 10 to 70% [14, 18]. Furthermore, the majority of studies of antibiotic de-escalation are limited to the intensive care setting and often to one disease entity such as ventilator associated pneumonia [1–12]. The frequency of hospital-wide antibiotic de-escalation in a setting with an established stewardship program has not been described. Thus, the purpose of this study was to measure the frequency of antibiotic de-escalation for one commonly used combination of empiric antibiotic therapy in an academic, tertiary care medical center with an established antimicrobial stewardship program (ASP) in order to describe a plausible de-escalation benchmark.

## Methods

### Study design

The study took place at Wake Forest Baptist Medical Center (WFBMC), an 885-bed tertiary care referral center with an active ASP. A list of 2,937 candidate patients ≥18 years of age was generated through a pharmacy database identifying inpatients who were prescribed the simultaneous combination of intravenous piperacillin/tazobactam and vancomycin from January to December 2011. This particular combination of antibiotics was used as inclusion criteria because in antibiotic utilization reports in previous years, it represented the most common broad spectrum empiric antibiotic regimen prescribed in the inpatient setting at WFBMC.

A sample of 20 patients per month was randomly selected from the candidate list using an online random number generator. If a study patient was admitted and initiated on piperacillin/tazobactam and vancomycin twice within the same month, only data from the first hospital admission was considered. Patients were stratified by month to account for seasonal variability in antibiotic use and inpatient prescriber differences. A retrospective chart review of the electronic medical record was subsequently performed on the selected patients.

Data collected included age, sex, hospital service, attending physician, documented indication for antibiotics, number of days on piperacillin/tazobactam and vancomycin, de-escalated antibiotic regimens, culture data, radiographic diagnoses, occurrence of antibiotic de-escalation at 24, 48, 72 and 96 h time points, length of stay, final clinical diagnosis, continuation of antibiotics after discharge, and in-hospital mortality. The primary outcome assessed was the frequency of antibiotic de-escalation at 72 h.

Antibiotic de-escalation in patients with both positive and negative bacterial cultures was quantified in order to evaluate the impact of culture data on the frequency of antibiotic de-escalation. Antibiotic de-escalation at 96 h was assessed to account for clinical decision making that may have occurred using culture data at 72 h incubation.

### Definitions

Antibiotic de-escalation was defined as the use of narrower spectrum antibiotics or the discontinuation of antibiotics after initiation of broad spectrum empiric antibiotic therapy. An antibiotic regimen change that resulted in narrowed coverage of a group of pathogens, yet also expanded coverage to include an additional group of pathogens, was not considered to represent an antibiotic de-escalation event. For example, while excluding anti-pseudomonal therapy, a transition from piperacillin/tazobactam to ertapenem was not considered a de-escalation event, as coverage of extended spectrum beta-lactamase producing organisms was incorporated. Similarly, an antibiotic change of one broad-spectrum class to another was not considered a de-escalation event. For example, if piperacillin/tazobactam was changed to cefepime, this was not considered to represent de-escalation even though anaerobic activity was dropped.

Similar to how previous studies have analyzed patients who were lost to follow up, patients were evaluated in the context of the unfavorable outcome group. Therefore, patients who expired within 72 h of initiation of broad spectrum antibiotics were considered not eligible for de-escalation and were therefore analyzed as not having their antibiotics de-escalated. These patients were subsequently excluded from analysis in determining a modified mortality rate. Patients who were discharged within 72 h had their antibiotic regimens analyzed analogous to those admitted for longer than 72 h.

The indication for antibiotic therapy was ascertained from documentation in the electronic medical record by the primary team at the time of simultaneous piperacillin/tazobactam and vancomycin use. Sepsis was recorded as the indication for antibiotics in patients who either had “sepsis” or “SIRS” documented in their medical record as the reason for initiation of antibiotics. Sepsis as a consequence of a known source (e.g. pneumonia) was considered as the source infection and not sepsis. Patients who had defined microbiological data from any source were considered to have positive cultures.

### Description of Antibiotic Stewardship Program

The ASP at WFBMC had been in place for 11 years at the time of the study. The core team consisted of two Infectious Diseases (ID) physicians acting as primary and associate medical directors, two ID PharmDs, an ID PharmD administrator, and an ID Pharmacy resident. The main components of the program during this entire time period were prospective audit and feedback (PAF) and preauthorization for selected antimicrobials. PAF was conducted 3 times per week by an ID PharmD in which patients were identified by an informatics generated list of positive sterile body fluids, use of selected antimicrobials, diagnosis of a selected infection, or referral by another clinical ward PharmD. Identified patient records were examined and improvements in antibiotic use were communicated back to the prescribing physician by phone. Antibiotic de-escalation, if appropriate, would be suggested at that time. The emphasis of PAF varied over the time period of the study depending on the ASP priority needs for intervention.

Antibiotics that had been selected for PAF at least one time during the period before or during the study included carbapenems, linezolid, aztreonam, oral vancomycin, and intravenous fluoroquinolones. Intravenous vancomycin or piperacillin/tazobactam was not selected for PAF specifically, but may have been included in an intervention when they were used for a scrutinized infection, or by other PharmD referral. Broad spectrum antibiotics that required pre-authorization included carbapenems, aztreonam, ceftazidime, linezolid, and daptomycin, but not cefepime, vancomycin or piperacillin/tazobactam.

De-escalation interventions other than PAF included ASP team suggestions for subsequent de-escalation at the time of pre-authorization, review of antibiotics by the ASP team or other clinical PharmD at the time of patient transfer (particularly intensive care unit transfer to the ward), ward or intensive care unit (ICU) clinical PharmD suggestion during daily rounds, quarterly housestaff case-based education, and ongoing provider education in large group presentations or at departmental meetings. Automatic stop orders or formal antibiotic “time-outs” were not used. Electronic medical record clinical decision support is not a component of the ASP, but electronic order sets that include antibiotics are available for commonly encountered infections. Local guidelines are available on the ASP website.

### Statistical analysis

Odds Ratios with 95% confidence intervals were calculated to determine differences between groups of categorical data. Quantile regression was performed to evaluate differences in hospital length of stay between patients whose antibiotics were de-escalated and not de-escalated.

## Results

Data was collected from 240 patients who were treated with the simultaneous combination of piperacillin/tazobactam and vancomycin during the study period. Patient age ranged from 19 to 94 years with a median age of 64 years. Antibiotic regimens were de-escalated in 151 (63%) and 175 (73%) patients by 72 and 96 h, respectively.

At 24, 48, 72, and 96 h, the percentage of patients who did not have their antibiotics de-escalated was 62, 43, 37, and 27% respectively. Of the patients whose antibiotic regimen was de-escalated, the greatest proportion occurred in the 0–24 h time period. Successive 24-h time increments reveal less dramatic proportions of de-escalation.

**Fig. 1:**
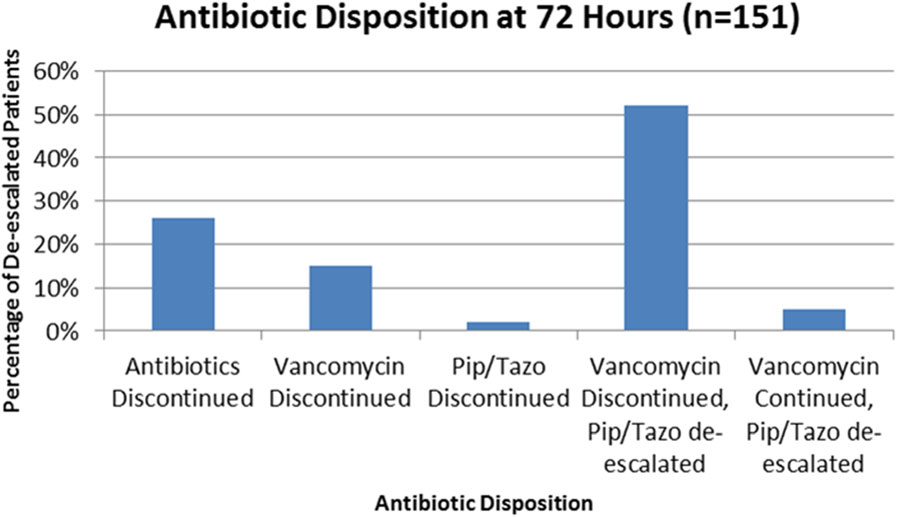
Disposition of piperacillin/tazobactam and vancomycin therapy at 72 h in patients who had their antibiotic regimens de-escalated

Antibiotic disposition at 72 h among patients who had their antibiotics de-escalated was heterogeneous (Fig. 1). Notably, for 79 (52%) patients who had their antibiotics de-escalated, vancomycin was discontinued and piperacillin/tazobactam was changed to an antibiotic with a narrowed spectrum. The most common antibiotics that were prescribed for de-escalation from piperacillin/tazobactam were moxifloxacin and ceftriaxone. Thirty-nine (26%) patients had their antibiotic therapy completely discontinued.

The most commonly documented indications for initiation of broad spectrum antibiotics were pneumonia, sepsis, and skin and soft tissue infections (Table 1). We observed the lowest de-escalation rates for skin and soft tissue infections (44%). While all other indications showed higher de-escalation rates, the difference was only statistically significant for urinary tract infections, Odds Ratio = 18.8 (95% CI: 2.2 – 162.9).

**Table 1:** Antibiotic indication and regimen de-escalation by 72 h

Antibiotic Indication	Total Study Population *n* = 240 (%)	Patients with regimen de-escalated by 72 h for each indication *n* = 151 (%)	Odds Ratio (95% CI)
Pneumonia	91 (38)	57 (63)	2.1 (0.9–5.0)
Sepsis	64 (27)	39 (61)	2.0 (0.8–4.9)
Skin or Soft Tissue Infection	27 (11)	12 (44)	Ref
Fever	23 (10)	14 (61)	1.9 (0.6–6.0)
Urinary Tract Infection	16 (6)	15 (94)	18.8 (2.2–162.9)
Other	19 (8)	14 (74)	3.5 (1.0–12.5)

Overall, 234 patients (98%) in the study population had microbiologic culture specimens obtained during their hospitalization. Of those, 94 (40%) patients had positive culture results. Antibiotic regimens were de-escalated by 96 h in 67 (71%) patients with positive culture results and in 101 (72%) patients with negative culture results. This difference was not statistically significant. A subsequent analysis was performed on patients with positive cultures whose regimens were not de-escalated (*n* = 27). Of these, 17 (63%) patients would have been appropriate for de-escalation based on their defined microbiologic data. Eight (30%) patients would have not been appropriate for de-escalation, and two (7%) died within 24 h, and consequently, would not have been eligible.

Patients admitted to oncology services had a higher frequency (71 vs. 63%) of antibiotic de-escalation at 72 h compared to the study population as a whole, although this was not statistically significant. Patients admitted to the remainder of services, including critical care units, had a similar frequency of antibiotic de-escalation to that observed in the overall study population (Table 2). The two most common documented indications for empiric vancomycin and piperacillin/tazobactam on critical care compared to oncology services were sepsis and pneumonia, and fever and pneumonia respectively (41 and 34 vs. 38 and 24%). Among other services, pneumonia and sepsis were the most common (41 and 23% respectively).

**Table 2:** Patients with antibiotic regimen de-escalated by service

Time (hours) or Odds Ratio (95% CI)	Total *n* = 240 (%)	Critical Care *n* = 58 (%)	Oncology *n* = 21 (%)	Other *n* = 161 (%)
24	90 (38)	28 (48)	5 (24)	57 (35)
48	136 (57)	31 (53)	9 (43)	96 (60)
72	151 (63)	36 (62)	15 (71)	100 (62)
96	175 (73)	40 (69)	17 (81)	118 (73)
Odds Ratio (95% CI) at 72 h	*N/A*	0.7 (0.2–1.9)	Ref	0.7 (0.2–1.8)

The median length of stay was 4 days shorter in patients who had their antibiotics de-escalated compared to those who did not (6 vs.10 days), *p* = 0.0003. One hundred fourteen (48%) patients were discharged home on antibiotics. The median length of stay for those patients was 5 days as compared to 9 days for those who were not discharged on antibiotics, *p* = 0.01.

Thirty-four (14%) study patients experienced in-hospital mortality. Mortality rates were significantly lower among patients who had their antibiotic regimens de-escalated compared to patients who did not, *p* = 0.002, Mortality Odds Ratio = 0.31 (95% CI: 0.14–0.65). However, 12 patients expired within 72 h of initiation of piperacillin/tazobactam plus vancomycin and were not eligible to have their antibiotic regimen de-escalated at 72 h. Therefore, a secondary analysis or modified mortality rate was also calculated to account for the impact these patients would have on the difference in mortality rates between groups (Table 3). In this analysis, only patients who were still living at 72 h were considered and no significant difference in mortality was observed between those whose antibiotic regimens were de-escalated and those whose regimens were not, Modified Mortality Odds Ratio = 0.84 (95% CI: 0.34–2.05).

**Table 3:** Patient mortality and length of stay with de-escalation

Patient Characteristics	Antibiotic regimen de-escalated *n* = 151 (%)	Antibiotic regimen not de-escalated *n* = 89 (%)	Odds Ratio (95% CI)	*p*-value
Mortality	13 (9)	21 (24)	0.31 (0.14–0.65)	0.002
Modified Mortality^a^	13 (9)	9 (10)	0.84 (0.34–2.05)	*NS*
Median Length of Stay^b^(days)	6	10	*N/A*	0.0003

^a^Modified Mortality: considers only patients who survived past day 3 and were subsequently eligible for antibiotic de-escalation at 72 h

^b^Overall Median LOS was 7 with an Interquartile Range of 4–13

## Discussion

This study of antibiotic de-escalation for one of the most commonly prescribed broad spectrum antibiotic regimens for empiric therapy, vancomycin plus piperacillin/tazobactam, showed that the majority of patients, approximately two-thirds, had their antibiotic regimens de-escalated by 72 h in a single institution with a well-established and resourced ASP. In addition, over half of patients had their regimens de-escalated by 48 h. The high proportion of patients de-escalated from the study antibiotics was reflected across a range of clinical services and presenting infections.

While the frequency with which hospital-wide antibiotic de-escalation occurs is not well-described in the literature, published estimates of antibiotic de-escalation are widely variable and range from 10 to 70% [14, 18]. In our study, antibiotic de-escalation occurred in nearly two-thirds of patients by 72 h. As antibiotic de-escalation is a key function of ASPs, including ours, the presence of an established ASP may facilitate higher rates of antibiotic de-escalation [19, 20]. Consequently, the frequency of de-escalation observed in this study is likely higher than that of a medical center that does not have an established ASP. Importantly, our study does not provide a measure of effectiveness of our ASP or compare the frequency of antibiotic de-escalation before-and-after the establishment of the ASP at our institution. Rather, it measures hospital-wide antibiotic de-escalation to provide a plausible benchmark for institutions with a multi-disciplinary ASP utilizing prospective audit and feedback and prior authorization as its core components.

Previous studies on antibiotic de-escalation are largely confined to the intensive care setting, are disease-specific, and highlight that de-escalation is safe and is not associated with worse outcomes. For example, in a randomized, prospective trial of 81 intensive care unit (ICU) patients with ventilator-associated pneumonia (VAP), *Singh* et al. found that patients whose antibiotic regimens were de-escalated were less likely to develop antibiotic resistant super-infections compared to those whose regimen was not de-escalated (15 vs 35%, *p* = 0.017) [1]. Similarly, a 2007 observational, prospective study involving 143 patients with VAP demonstrated decreased mortality at day 15 (5 vs 32%) and day 28 (12 vs. 44%) with shorter ICU and hospital stays in patients whose antibiotic regimens were de-escalated [2].

A number of study observations warrant further discussion. First, patients admitted to oncology services were observed to have the highest frequency of antibiotic de-escalation. While the high proportion of de-escalation in this population is surprising, the oncology patients captured in this study comprised a minority of the study population and cefepime, not piperacillin/tazobactam, plus/minus vancomycin, were the fever-neutropenia protocol indicated antibiotics. Although fever was the most common documented indication for broad spectrum antibiotics on oncology services, only two of these patients had concurrent neutropenia documented. Additionally, while hyperpyrexia in immunocompromised patients may have led prescribers to utilize empiric broad spectrum antimicrobial therapy, they were also quick to de-escalate therapy which may reflect recognition of limited instances where anti-MRSA therapy was indicated, even if broad spectrum gram negative coverage was.

Next, we did not observe significant differences in the proportion of patients de-escalated when they were stratified according to the documented indication for antibiotics, with the exception of urinary tract infections. The higher frequency of antibiotic de-escalation in patients with urinary tract infections (94%) compared to the overall study population is likely related to several different factors. First, patients with urinary tract infections represented a very small percentage of the total study population (7%). Additionally, piperacillin/tazobactam plus vancomycin is not a regimen that is typically used for the empiric treatment of urinary tract infections. Thirdly, culture results are often available for these patients by 48 h. Thus, prescribers are likely to de-escalate these regimens quickly.

The vast majority of patients in our study had culture data available to assist prescribers with decision-making. However, no appreciable difference was observed in the frequency of de-escalation between patients who had positive cultures when compared to patients with negative cultures (71 and 72% respectively). While the majority of patients with positive cultures were de-escalated, a secondary analysis of patients with positive cultures whose regimens were not de-escalated revealed that 17 (63%) would have been appropriate for de-escalation based on defined microbiologic isolates. In most cases, the presence of culture data, either positive or negative, may facilitate de-escalation of antibiotic therapy, but further study is needed to determine how clinicians use culture data in their de-escalation practice. While positive cultures can define microbiology and allow prescribers to tailor antibiotic regimens to the isolated organisms, negative cultures may provide reassurance for prescribers as they de-escalate antibiotic regimens. The ultimate decision to de-escalate therapy is likely influenced by an interplay of dynamic clinical variables that may be further compounded by severity of illness and complex susceptibility patterns.

Another interesting finding is the large proportion of patients whose antibiotic regimen was de-escalated within 24 h. The decision to de-escalate in this timeframe is likely not based on culture results, but instead on the patient’s clinical status and the decision that the patient’s clinical picture did not warrant the use of broad spectrum antimicrobial therapy.

Finally, observed mortality rates and hospital length of stay were both lower in patients who had their antibiotics de-escalated compared to those who did not. However, these associations may be surrogate markers of overall clinical status and the severity of illness, which were not controlled for in this study. Nevertheless, our data highlights that de-escalation was not associated with an increase in mortality.

Limitations of this study include the retrospective study design and the reliance on medical record documentation for data collection, including the indications for empiric vancomycin and piperacillin/tazobactam. Certain patient populations were also under-represented in this study, such as oncology patients and patients with urinary tract infections. This study measured the frequency of antibiotic de-escalation at a single center with an established ASP, and as a result, may not be applicable to all institutions. Additionally, only patients who were empirically started on piperacillin/tazobactam and vancomycin were included in the study. Consequently, we did not measure all antibiotic de-escalation that may have occurred during the study period and frequency of de-escalation may differ for other broad spectrum empiric antibiotic regimens. Finally, this study did not evaluate the appropriateness of antibiotics including both empiric and de-escalated regimens.

## Conclusions

De-escalation of empiric antibacterial therapy is increasingly recognized as an important principle of antibiotic stewardship. While antibiotic de-escalation may not be feasible or appropriate in every instance, this study adds to the literature as potential benchmarks for antibiotic de-escalation are being considered. Future studies should examine the frequency of antibiotic de-escalation at other institutions both with and without established ASPs. In addition, longitudinal comparisons of the frequency of antibiotic de-escalation may add important information on whether frequencies change over time at a given institution. While our study measured the frequency of antibiotic de-escalation, we did not assess appropriateness of either the empiric or de-escalated antibiotic regimen. Interpreting the occurrence of antibiotic de-escalation in the context of appropriateness would assist in determining whether a 72-h antibiotic de-escalation frequency of 60–70% is a reasonable benchmark.

## Abbreviations

ASP: Antimicrobial stewardship program
WFBMC: Wake forest baptist medical center
ID: Infectious diseases
PAF: Prospective audit and feedback
ICU: Intensive care unit
VAP: Ventilator-associated pneumonia
